# SARS-CoV-2 seroprevalence and extent of infection under-ascertainment in seven regions of Uzbekistan: a population-based serosurvey, May–July 2022

**DOI:** 10.3389/fpubh.2026.1821072

**Published:** 2026-09-29

**Authors:** Aybek Khodiev, Alexander J. Millman, Botirjon Kurbanov, Emily Carter, Michelle Sloan, Zulfiya Abdurakhimova, Shakhida Karamatova, Adkhamjon Mamatkulov, Elizaveta Joldasova, Dilafkor Mirdjalilov, Leyla Koroli, Nataliya Kan, Abdulaziz Abdurasulov, Erkin Musabaev

**Affiliations:** 1 CDC Uzbekistan Country Office, Tashkent, Uzbekistan; 2 CDC Central Asia Regional Office, Almaty, Kazakhstan; 3 Committee for Sanitary and Epidemiological Welfare and Public Health, Ministry of Health of the Republic of Uzbekistan, Tashkent, Uzbekistan; 4 National Center for Immunization and Respiratory Diseases, Centers for Disease Control and Prevention, Atlanta, GA, United States; 5 Research Institute of Virology, Ministry of Health of the Republic of Uzbekistan, Tashkent, Uzbekistan

**Keywords:** complex survey design, COVID-19 vaccination, population-based survey, SARS-CoV-2, seroprevalence, surveillance evaluation, Uzbekistan

## Abstract

Accurate estimation of SARS-CoV-2 transmission was limited in many settings by incomplete diagnostic testing and case reporting. We conducted a population-based cross-sectional serosurvey in seven regions of Uzbekistan between May and July 2022 to estimate SARS-CoV-2 seroprevalence, describe self-reported vaccination coverage, and assess the implications for routine surveillance. A two-stage cluster sampling design was used to randomly select polyclinic catchment areas and registered residents aged ≥5 years. Survey weights accounted for selection probabilities, non-response, and regional population structure. Among 15,000 invited individuals, 13,173 completed both questionnaire and serological testing, corresponding to an 87.8% response rate. Weighted seroprevalence was 98.3% (95% CI 98.0–98.6), indicating extensive cumulative exposure through infection, vaccination, or both. Self-reported vaccination coverage was 59.0% among adults and substantially lower among children. Approximately 243,000 COVID-19 cases had been officially reported nationally by August 2022; however, this national count is not directly comparable with seroprevalence in the seven surveyed regions. Nevertheless, the high seroprevalence, including among groups with low vaccination coverage, is suggestive of substantial transmission not identified by routine case reporting. Population-based serosurveys can therefore complement routine surveillance when interpreted alongside vaccination and epidemiological data.

## Introduction

Severe acute respiratory syndrome coronavirus 2 (SARS-CoV-2), the causative agent of COVID-19, emerged in late 2019 and rapidly progressed into a global pandemic. By February 2023, more than 700 million cases and nearly 7 million deaths had been reported worldwide (1). However, limited diagnostic testing, asymptomatic infections, healthcare-seeking behavior, false-negative results, and incomplete reporting resulted in underestimation of the true burden of infection (2). Consequently, surveillance systems based solely on laboratory-confirmed cases may not fully describe the geographic spread or cumulative impact of the pandemic (3).

Uzbekistan implemented nationwide public health measures beginning in February 2020, and the first laboratory-confirmed COVID-19 case was reported on March 15, 2020. By August 2022, approximately 243,000 confirmed cases had been officially registered (4). Population-based serosurveys provide an important complementary approach to routine surveillance by estimating cumulative exposure to infection and vaccination at the population level (5, 6).

Prior to the availability of vaccination, a national serological survey conducted in Uzbekistan in August–September 2020 estimated SARS-CoV-2 antibody prevalence at 23%, suggesting widespread and regionally heterogenous transmission above the reported case numbers (7). Following continued epidemic spread and the introduction of COVID-19 vaccination in March 2021, we conducted a large population-based SARS-CoV-2 serosurvey in seven geographically diverse regions of Uzbekistan during May–July 2022. The survey included 13,173 participants aged ≥5 years from regions collectively accounting for approximately 57% of the national population and representing a range of prior SARS-CoV-2 seroprevalence levels. We aimed to estimate regional and demographic patterns of SARS-CoV-2 seroprevalence, describe self-reported vaccination coverage, and assess the implications of population-level serologic findings for interpretation of routine COVID-19 surveillance.

## Materials and methods

### Study design and sampling

This serosurvey was designed according to the World Health Organization (WHO) UNITY protocol (8). A two-stage cluster randomized sampling approach was used to obtain population-representative estimates within each participating region. Because implementation of the serosurvey in all 14 administrative regions was not feasible because of logistical and resource constraints, seven regions were selected in coordination with the Ministry of Health of Uzbekistan. Selection considered population size, geographic distribution, and regional SARS-CoV-2 antibody prevalence observed in the 2020 serosurvey. Based on the prevalence categories used in that survey, Andijan Region represented a low-seroprevalence setting (6.04%); Kashkadarya Region (17.03%), Tashkent City (20.72%), Tashkent Region (24.15%), and Samarkand Region (31.03%) represented moderate-seroprevalence settings; and the Republic of Karakalpakstan (32.02%) and Khorezm Region (36.02%) represented high-seroprevalence settings. The selected regions collectively accounted for approximately 57% of Uzbekistan’s population and included Andijan Region in the eastern Fergana Valley, Tashkent City and Tashkent Region, Samarkand Region in central Uzbekistan, Kashkadarya Region in the south, and Khorezm Region and the Republic of Karakalpakstan in the west and northwest. Because the seven regions were not randomly selected from all 14 administrative regions, the resulting estimates are representative of the populations covered by the sampling frames within the participating regions and should not be interpreted as nationally representative estimates for Uzbekistan. The sampling framework was adapted to the national polyclinic-based primary healthcare system, in which residents are administratively registered within defined polyclinic catchment areas. In the first stage, polyclinics were randomly selected; in the second stage, participants were randomly selected from the population rosters of the selected polyclinics.

Polyclinic rosters are intended to enumerate residents assigned according to their registered place of residence. Accordingly, the sampling frame represented residents recorded in the selected polyclinic catchments. Persons without current registration, temporary or mobile populations, residents of institutions not included in polyclinic rosters, and persons unable to attend the designated survey sites may have been underrepresented.

Eligible participants were persons aged 5 years and older residing in the catchment areas of selected polyclinics. The pediatric stratum was defined as children aged 5–17 years, consistent with the prespecified combined SARS-CoV-2, HBV, and HCV serosurvey protocol; findings from the HBV and HCV components are not presented in this manuscript. Children younger than 5 years were excluded.

In the first stage, polyclinic catchments were selected as primary sampling units (PSUs) from regional sampling frames stratified by region and urban–rural residence, with allocation proportional to population size and selection by simple random sampling without replacement. In the second stage, registered residents were randomly selected from polyclinic population rosters, including children aged 5–17 years and adults aged ≥18 years. A total of 100 PSUs (50 urban and 50 rural) were selected across the study regions; Tashkent City included only urban strata. All selected PSUs contained eligible participants, and no replacements were required (Table 1).

**Table 1:** Distribution of selected primary sampling units (PSUs) and targeted sample size by region and urban–rural stratum.

Region	Urban PSUs (selected/total)	Rural PSUs (selected/total)	Total selected PSUs	Targeted sample
Andijan	8/10	8/313	16	2,400
Kashkadarya	7/23	10/244	17	2,550
Khorezm	3/5	6/170	9	1,350
Samarkand	7/14	13/168	20	3,000
Republic of Karakalpakstan	5/8	5/186	10	1,500
Tashkent Region	7/17	8/259	15	2,250
Tashkent City	13/62	—	13	1,950
Total	50/139	50/1,340	100	15,000

The target sample size was approximately 15,000 participants (5,000 children and 10,000 adults), calculated assuming 25% seroprevalence, 95% response rate, a design effect of 2, and ±1% precision (9, 10). The pediatric sample size was expanded to support a concurrent hepatitis B serosurvey not reported here.

### Data collection and laboratory testing

The survey was conducted between May and July 2022. Selected participants attended designated polyclinic sites, where verbal informed consent was obtained for participation (parental verbal consent for participants aged <18 years). Participants completed a standardized questionnaire capturing demographic characteristics, COVID-19 history, symptoms, and self-reported vaccination status, and provided blood samples for serological testing.

The collected blood samples were transported to the virology laboratory at the Institute of Virology, Ministry of Health of Uzbekistan, where serum samples were tested for SARS-CoV-2 IgG antibodies using the D-5501 SARS-CoV-2-IgG-EIA-BEST enzyme-linked immunosorbent assay (Vector-Best, Novosibirsk, Russia; sensitivity 98.9% [97.2–98.9%] and specificity 100.0% [95.1–100.0%]). The assay detects IgG antibodies directed against the SARS-CoV-2 spike (S) protein. Because anti-S antibodies may result from either natural SARS-CoV-2 infection or vaccination, the assay could not distinguish infection-induced from vaccine-induced antibodies. RT-PCR testing was not performed as part of this serosurvey; SARS-CoV-2 exposure was assessed using serologic detection of IgG antibodies.

### Weighting and statistical analysis

Survey weights were constructed to account for unequal probabilities of selection, stratified sampling, and non-response. Final weights incorporated inverse probabilities of PSU and individual selection, adjustment for stratum-specific response rates, and post-stratification calibration to 2022 regional population distributions obtained from the State Committee of the Republic of Uzbekistan on Statistics (11). Weighted estimates were intended to represent the population residing in the seven surveyed regions of Uzbekistan.

Response rates were calculated separately within region-by-urban/rural-by-age strata, with age categorized as children aged 5–17 years and adults aged ≥18 years. Within each stratum, the response rate was calculated as the number of selected persons with both questionnaire and laboratory results divided by the number selected for participation. The non-response adjustment factor was the inverse of the corresponding stratum-specific response rate. Final weights were calculated as the product of the stage-one PSU selection weight, stage-two individual selection weight, non-response adjustment factor, and post-stratification calibration factor.

Analyses accounted for the complex survey design using the *survey* package in R (version 4.2.1) (12). Variance estimation employed jackknife replicate weights generated within sampling strata (13). Weighted seroprevalence and vaccination coverage were estimated overall and by demographic characteristics. Associations with seropositivity were evaluated using survey-weighted logistic regression models adjusted for age, sex, residence, and region. Statistical analyses were conducted using R and RStudio (14). Survey-weighted regression models were fitted using the same replicate-weight survey design. Consequently, standard errors and confidence intervals incorporated sampling weights, stratification, and clustering at the PSU level.

Variance estimation used 100 jackknife replicate weights, corresponding to the 100 selected PSUs. Each replicate omitted one PSU within its sampling stratum and rescaled the weights of the remaining PSUs in that stratum. The design contained 13 region-by-urban/rural sampling strata, and every stratum contained multiple PSUs.

Because seropositivity was nearly universal, odds ratios may appear large despite small absolute differences in seroprevalence and should not be interpreted as prevalence ratios. The regression findings were therefore considered exploratory, and weighted prevalence estimates with confidence intervals remained the primary results.

### Patient and public involvement

Patients and members of the public were not involved in the design, conduct, reporting, or dissemination of this study. The survey was implemented as part of an emergency public health response using the WHO UNITY protocol and existing national surveillance infrastructure. Participants contributed solely as survey respondents and sample donors.

## Results

Among the 15,000 participants invited, 13,727 consented to participate in the survey and 13,173 responded to the questionnaire and provided a blood sample (9,080 adults and 4,093 children; 87.8% response rate). Participant inclusion is summarized in Figure 1. The demographic characteristics are shown in Table 2.

**Figure 1:**
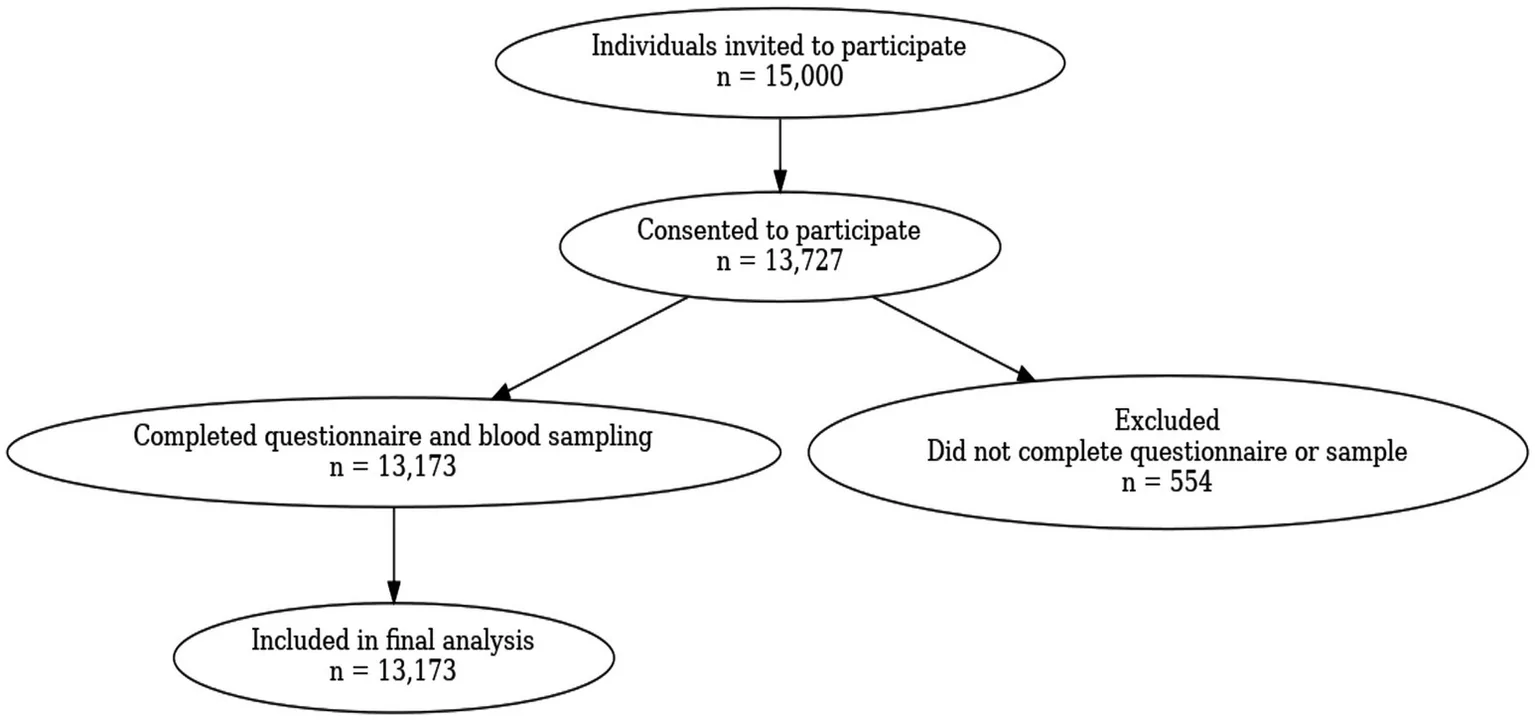
Participant flow diagram showing recruitment, participation, and inclusion in the final analysis of the SARS-CoV-2 serosurvey conducted in seven regions of Uzbekistan, May–July 2022.

**Table 2:** Seroprevalence by region, age group, urban status, and sex.

Characteristic	Positive/total, *N*	Seroprevalence–weighted % (95% CI)	OR (95%CI)	*p*-value	AdjOR (95% CI)	Adj *p*-value
Region						
Andijan region	2176/2216	98.1 (96.6–99.0)	Ref		Ref	
Republic of Karakalpakstan	1375/1391	99.2 (98.8–99.5)	2.4 (1.1–5.0)	0.025	2.4 (0.9–6.2)	0.060
Kashkadarya region	2011/2047	98.5 (97.3–99.2)	1.3 (0.5–3.1)	0.561	1.4 (0.6–3.5)	0.414
Khorezm region	1215/1240	98.1 (96.8–98.9)	1.0 (0.4–2.2)	0.980	1.0 (0.4–2.2)	0.971
Samarkand region	2188/2238	98.0 (97.2–98.5)	0.9 (0.5–1.9)	0.813	1.1 (0.5–2.1)	0.866
Tashkent city	1807/1842	98.5 (96.9–99.3)	1.3 (0.5–3.4)	0.635	1.3 (0.5–3.3)	0.583
Tashkent region	2163/2199	98.3 (97.7–98.8)	1.1 (0.6–2.3)	0.708	1.2 (0.6–2.4)	0.518
Age group						
5–9	1652/1731	94.3 (91.9–96.0)	Ref		Ref	
10–14	1557/1600	97.7 (96.5–98.5)	2.6 (1.4–5.0)	0.004	2.6 (1.4–4.9)	0.004
15–17	752/762	98.8 (97.3–99.5)	4.9 (2.3–10.8)	<0.001	5.2 (2.4–11.2)	<0.001
18–29	1884/1900	99.3 (98.5–99.6)	8.0 (3.6–18.0)	<0.001	8.1 (3.6–18.4)	<0.001
30–39	2330/2362	98.8 (98.1–99.2)	5.0 (2.8–9.0)	<0.001	4.8 (2.7–8.8)	<0.001
40–49	1766/1778	99.5 (98.9–99.8)	12.6 (5.0–32.0)	<0.001	12.1 (4.9–30.2)	<0.001
50–59	1532/1546	99.0 (98.0–99.5)	6.2 (2.8–14.0)	<0.001	5.9 (2.7–13.2)	<0.001
60+	1462/1494	98.4 (97.2–99.0)	2.18 (1.44–3.31)	<0.001	3.6 (1.9–6.9)	<0.001
Residence						
Urban	6823/6941	98.3 (98.2–98.5)	Ref		Ref	
Rural	6112/6232	98.0 (97.9–98.2)	0.9 (0.6–1.4)	0.707	1.0 (0.7–1.4)	0.977
Sex						
Male	5129/5255	97.9 (97.2–98.5)	Ref		Ref	
Female	7806/7918	98.6 (98.3–98.9)	1.6 (1.0–2.3)	0.032	1.3 (0.9–1.9)	0.136
Total	12,935/13173	98.3 (98.0–98.6)				

OR, odds ratio; AdjOR, adjusted odds ratio (adjusted for age group, sex, and residence status); Ref, reference category; 95% CI, 95% Confidence Interval.

Seroprevalence of SARS-CoV-2 antibodies among all people surveyed was estimated to be 98.3% (95% CI: 98.0–98.6) and was similarly high in all regions. The Republic of Karakalpakstan exhibited the highest seroprevalence at 99.2% (95% CI: 98.8–99.5%), while Samarkand Region reported the lowest at 98.0% (95% CI: 97.2–98.5%). The children’s 5–9 age group had a significantly lower prevalence than each of the other age groups (Table 2). Seroprevalence among those ≥60 years was 98.4% (95% CI: 97.2–99.0), with self-reported vaccination coverage of 56.6% (95% CI: 49.8–63.2) (Table 3).

**Table 3:** Self-reported vaccination status of the responders by age group and region.

Characteristic	Vaccinated/total, *n*	% (95% CI)	AdjOR (95% CI)	*p*-value
Age group, years				
Children				
5–9	59/1553	3.3 (1.9–5.8)	Ref	
10–14	97/1424	5.8 (3.5–9.4)	1.8 (1.0–3.1)	0.048
15–17	127/674	17.4 (12.7–23.3)	6.1 (3.5–10.7)	<0.001
Subtotal	283/3651	6.9 (4.9–9.7)		
Adult				
18–29	894/1781	50.3 (42.8–57.8)	Ref	
30–39	1365/2222	60.6 (55.6–65.4)	1.5 (1.2–1.9)	0.001
40–49	1074/1691	64.0 (59.3–68.4)	1.8 (1.4–2.2)	<0.001
50–59	943/1463	64.1 (59.3–68.7)	1.8 (1.4–2.3)	<0.001
60+	835/1405	56.6 (49.8–63.2)	1.3 (1.0–1.7)	0.054
Subtotal	5111/8562	59.0 (54.2–63.7)		
Region				
Andijan region	1009/2047	49.9 (37.8–62.0)	Ref	
Republic of Karakalpakstan	751/1383	41.4 (30.2–53.6)	1.4 (0.7–2.8)	0.335
Kashkadarya region	664/1827	57.9 (47.8–67.3)	0.7 (0.4–1.4)	0.32
Khorezm region	619/1218	45.4 (42.0–48.8)	1.2 (0.7–2.0)	0.492
Samarkand region	734/1821	62.4 (49.7–73.9)	0.6 (0.3–1.2)	0.161
Tashkent City	664/1726	58.0 (53.7–62.3)	0.7 (0.4–1.2)	0.218
Tashkent region	953/2191	53.1 (44.4–61.5)	0.9 (0.5–1.6)	0.675
Total	5394/12213	53.5 (49.6–57.3)		

AdjOR, adjusted odds ratio (adjusted for age group, sex, and residence status); Ref, reference category; 95% CI, 95% Confidence Interval.

The odds of self-reported vaccination significantly increased for the 15–17 years age group compared to the 5–9 years age group (reference group), with an OR of 6.1 (95% CI: 3.5–10.7), *p*-value <0.001. Vaccination rates increased with age, with more than half of adults in each age group reporting vaccination. The odds of vaccination increased with age up to the 50–59-years age group and declined among adults aged ≥60 years. The 30–39, 40–49 and 50-59-years age groups have significantly higher odds of being vaccinated compared to the 18–29 years age group (reference group), both with a *p*-value of <0.001. Vaccination rates varied across different regions, with the highest rate (62.4%; 95% CI: 49.7–73.9) in the Samarkand region and the lowest rate (41.4%; 95% CI: 30.2–53.6) in the Republic of Karakalpakstan. The highest frequency of self-reported vaccination was found in the Samarkand region; however, no significant differences in vaccination between regions were found (Table 3).

## Discussion

By August 2022, approximately 243,000 COVID-19 cases had been officially reported in the total population of Uzbekistan. National reported cases were identified through routine diagnostic surveillance, including RT-PCR-based testing, whereas the present serosurvey measured cumulative anti-SARS-CoV-2 IgG seropositivity. This national case count is not directly comparable with the seroprevalence estimate because it included all age groups in the national population, whereas the serosurvey included persons aged ≥5 years from seven of Uzbekistan’s 14 regions, and because detected antibodies could have resulted from infection, vaccination, or both. The comparison should therefore be interpreted as contextual rather than as a direct estimate of the national infection-to-case ratio. Nevertheless, the very high seroprevalence in the surveyed regions, particularly among younger groups with low reported vaccination coverage, is consistent with substantial SARS-CoV-2 transmission that was not identified through routine case reporting.

With 13,173 participants across seven geographically and epidemiologically diverse regions, this study provides a large population-based assessment of SARS-CoV-2 seroprevalence in Uzbekistan and documented very high weighted seroprevalence (98.3%) by mid-2022. The substantial increase in seroprevalence compared with the August–September 2020 national survey (23.1%) likely reflects cumulative epidemic transmission together with the impact of vaccination introduced in March 2021. Similar increases in population seroprevalence following successive pandemic waves have been reported in Sweden (15), Mongolia (16), and the Dominican Republic (17). These country-specific findings are consistent with broader global seroprevalence patterns documented through the SeroTracker initiative (18). Confirmed case counts alone underestimate the true magnitude of SARS-CoV-2 transmission, particularly in settings with limited testing capacity or high proportions of asymptomatic infection (19).

By August 2022, national data indicated that approximately 58% of Uzbekistan’s total population had received at least one COVID-19 vaccine dose (20). In the present survey, self-reported vaccination coverage among adults aged ≥60 years was 56.6%; however, these estimates are not directly comparable because they refer to different populations and data sources. Coverage among adults aged ≥60 years was lower than that observed in several middle-aged groups. Given their increased risk of severe disease, maintaining adequate vaccination coverage in this population remains important (21). The survey did not collect information on reasons for non-vaccination; therefore, the relative contributions of access barriers, vaccine confidence, contraindications, or reporting error could not be assessed. Nevertheless, the finding underscores the importance of routinely monitoring vaccination uptake by age and risk group and using vaccination registries to identify coverage gaps (22). Such monitoring could support targeted communication, outreach, and booster-delivery strategies during future COVID-19 and other respiratory disease vaccination campaigns.

The observed discrepancy between serological estimates and reported case counts underscores the limitations of relying solely on routine surveillance data to assess epidemic magnitude. Population-based serosurveys provide complementary evidence by capturing cumulative exposure and seroprevalence patterns that are not reflected in case reporting systems. The value of seroepidemiological surveillance extends beyond SARS-CoV-2; population-level poliovirus serological studies have demonstrated how antibody data can identify potentially susceptible subgroups and inform vaccination and surveillance priorities (23). Such data are particularly valuable for interpreting epidemic trends, estimating remaining population susceptibility, and guiding vaccination and preparedness strategies.

Periodic serosurveys could be incorporated into an integrated respiratory surveillance framework rather than used as stand-alone activities. Surveys conducted at predefined intervals or after major epidemic waves could be stratified by age, region, and vaccination status and interpreted alongside routine case reporting, sentinel influenza-like illness and severe acute respiratory infection surveillance, hospitalization and mortality data, vaccination registries, and genomic surveillance. Linked vaccination records would improve differentiation of vaccine-related and infection-related antibody patterns, while genomic and sentinel surveillance would provide more timely information on circulating variants and current disease activity. This triangulated approach could help calibrate under-ascertainment in case reporting, identify demographic or geographic coverage gaps, and inform preparedness planning.

Several limitations should be considered when interpreting these findings. Because the survey included seven of the country’s 14 administrative regions, the findings should not be interpreted as fully nationally representative, although the selected regions accounted for approximately 57% of Uzbekistan’s population and were chosen to capture geographic and epidemiologic diversity. Although sampling was probabilistic within each included region, the seven regions themselves did not constitute a probability sample of the entire country. The estimates should therefore not be generalized to all of Uzbekistan, and epidemiologic patterns may have differed in regions that were not surveyed. Second, the cross-sectional design did not allow assessment of infection timing, antibody waning, or temporal changes in population seroprevalence. Third, the serologic assay could not distinguish infection-induced from vaccine-induced antibodies, limiting attribution of seropositivity to prior infection or vaccination. Vaccination status was self-reported and may therefore be subject to recall or reporting bias. In addition, although response rates were high and survey weighting was applied, residual non-response bias cannot be completely excluded. Immunocompromising conditions were not specifically assessed; because some immunocompromised persons may have reduced or absent detectable antibody responses following infection or vaccination, serology may underestimate prior exposure in this subgroup. Nevertheless, the probabilistic sampling design, standardized WHO UNITY methodology, and application of complex survey weights support the representativeness of estimates within the included regions.

## Conclusion

This population-based serosurvey found high SARS-CoV-2 seroprevalence across the seven surveyed regions of Uzbekistan by mid-2022, reflecting cumulative exposure through infection, vaccination, or both. The consistently high antibody prevalence across demographic and geographic groups, including groups with low reported vaccination coverage, is consistent with extensive transmission that was not fully captured by routine case-based surveillance. These findings support the use of periodic serosurveys alongside case reporting, vaccination registries, sentinel respiratory surveillance, and genomic surveillance to improve interpretation of epidemic patterns and strengthen preparedness for future respiratory epidemics.

## Data Availability

The datasets analyzed in this study are available from the Ministry of Health of the Republic of Uzbekistan upon reasonable request and subject to ethical approval. Requests to access the datasets should be directed to drmusabaev1956@gmail.com.
